# The evolution of nutrition management in children with severe neurological impairment with a focus on cerebral palsy

**DOI:** 10.1111/jhn.13277

**Published:** 2024-01-09

**Authors:** Stina Oftedal, Siobhan McCormack, Richard Stevenson, Katherine Benfer, Roslyn N. Boyd, Kristie Bell

**Affiliations:** ^1^ Queensland Cerebral Palsy Rehabilitation Research Centre, Faculty of Medicine The University of Queensland Child Health Research Centre Brisbane Queensland Australia; ^2^ Department of Child Development and Neurodisability Children's Health Ireland at Tallaght Dublin Ireland; ^3^ Department of Paediatrics, School of Medicine University of Galway Galway Ireland; ^4^ Division of Neurodevelopmental and Behavioral Pediatrics, Department of Pediatrics, School of Medicine University of Virginia Charlottesville Virginia USA; ^5^ Dietetics and Food Services Children's Health Queensland South Brisbane Queensland Australia

**Keywords:** blended food, cerebral palsy, disability, growth, malnutrition, tube feeding

## Abstract

Nutritional management of children with severe neurological impairment (SNI) is highly complex, and the profile of this population is changing. The aim of this narrative review was to give the reader a broad description of evolution of the nutritional management of children with SNI in a high resource setting. In the last decade, there has been an emphasis on using multiple anthropometric measures to monitor nutritional status in children with SNI, and several attempts at standardising the approach have been made. Tools such as the Feeding and Nutrition Screening Tool, the Subjective Global Nutrition Assessment, the Eating and Drinking Ability Classification System and the Focus on Early Eating and Drinking Swallowing (FEEDS) toolkit have become available. There has been an increased understanding of how the gut microbiome influences gastrointestinal symptoms common in children with SNI, and the use of fibre in the management of these has received attention. A new diagnosis, ‘gastrointestinal dystonia’, has been defined. The increased use and acceptance of blended food tube feeds has been a major development in the nutritional management of children with SNI, with reported benefits in managing gastrointestinal symptoms. New interventions to support eating and drinking skill development in children with SNI show promise. In conclusion, as the life expectancy of people with SNI increases due to advances in medical and nutrition care, our approach necessitates a view to long‐term health and quality of life. This involves balancing adequate nutrition to support growth, development and well‐being while avoiding overnutrition and its associated detrimental long‐term effects.

## Key points

- Earlier diagnosis and longer life expectancy have changed the profile of children with severe neurological impairment.
- Newer assessment tools available support a more holistic nutritional status assessment.
- Many international societies actively promote the use of blended food tube feeds.
- Awareness of the importance of the gut microbiome has driven an interest in fibre.
- A shared decision‐making approach between parents/caregivers and healthcare practitioners is considered best practice.

## Graphical abstract

High‐quality nutritional management in children with severe neurological impairment is highly complex and critical for their development and well‐being. The last decade has seen an emphasis on tracking multiple anthropometric measures, development of new tools, an increased interest in using fibre in gastrointestinal symptom management, parent demand for blended food tube feeds and awareness of supporting healthy ageing.

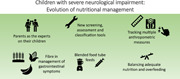

## INTRODUCTION

Severe neurological impairment (SNI) is a term used to describe a group of more than 400 chronic, heterogeneous conditions which share a core feature of disorder of the central nervous system.^1^, ^2^, ^3^ It arises in utero or throughout the early developmental period and results in motor impairments, cognitive impairments and medical complexity, resulting in functional limitations to activities of daily living.^4^ Children with SNI would typically be classified as Gross Motor Function Classification System (GMFCS) level IV or V and primarily use a wheelchair for mobility and function.^5^ Impairments are permanent but may be either progressive or static.^2^

**Figure 1:**
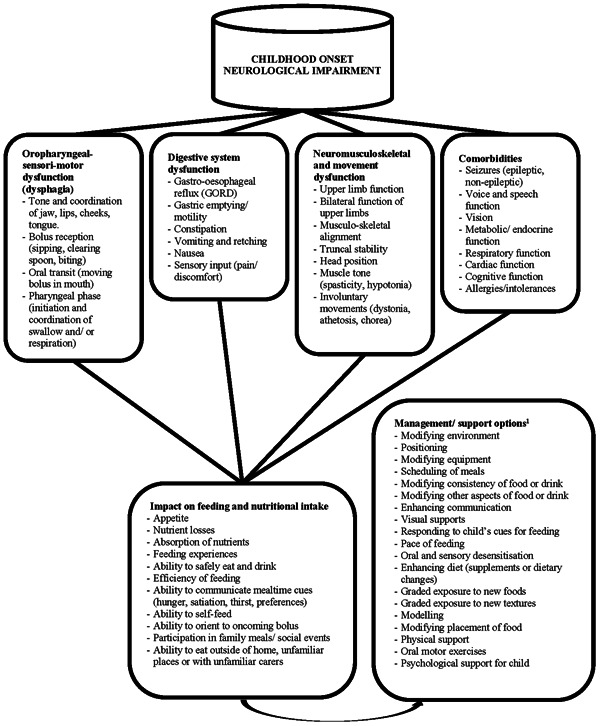
The possible factors impacting feeding and nutritional intake in children with severe neurological impairment.^1^ From the Focus on Early Eating, Drinking and Swallowing (FEEDS) toolkit of interventions.^9^

The central nervous system controls feeding and swallowing through complex pathways involving cranial nerve nuclei, the brainstem and cortex.^6^ Safe feeding and swallowing rely on a multitude of sensory inputs being correctly registered, interpreted and communicated onwards to create motor signals to orchestrate oral processing of the bolus, swallowing and respiration.^7^ Atypical development of the central nervous system may impact not only the oral‐sensorimotor apparatus itself but also gross motor control of the head, neck and trunk, ability to self‐feed, vision and cognition. These factors impact a child's ability to consume adequate nutrition safely and efficiently, placing them at significant risk of malnutrition. Further, the central nervous system modulates the enteric nervous system, which controls important functions like motility, secretion and gastrointestinal blood flow. In children with neurological impairment, this interaction can be disordered and may manifest as gastroesophageal reflux, vomiting or constipation.^8^ Consequent pain, discomfort, nutrient loss and negative associations related to feeding may also place a child at risk of poor nutritional status.^6^ The possible impacts on aspects related to feeding and nutritional intake are summarised in Figure 1.

The profile of children with SNI is changing. They are being identified earlier, and in many cases a diagnosis of cerebral palsy (CP) can now be made before the age of 6 months,^10^, ^11^ which enables intervention at a time of high neural plasticity to maximise outcomes.^12^, ^13^ Increased survival following preterm birth,^14^ congenital anomalies,^15^ and improvements in intensive care^16^ and healthcare in general have contributed to an increased number of children with SNI and increased life expectancy.^17^, ^18^, ^19^, ^20^ This necessitates a lifespan approach with consideration of medical and nutrition management which preserves health as much as possible into later life.

Appropriate nutritional management in this population is highly complex and critical for their development and well‐being. Parents and caregivers are now more easily able to access information and peer‐support around the nutritional management of their children through the internet and social media. This has enabled parents and caregivers to be more active, informed participants in goal setting and treatment decisions but may also increase the need for clinician guidance in investigating and understanding the relevance and appropriateness of available treatments for their child. This narrative review aimed to examine the evolution of nutritional management of children with SNI in a high‐resource setting. Because most of the research in this area is on children with CP, the research referenced primarily reports on children with CP classified as GMFCS levels IV and V.

## NUTRITIONAL ASSESSMENT AND MONITORING

### Nutrition assessment

There is increased awareness of the importance of incorporating multiple measures to monitor nutritional status, and several attempts at standardising the approach to monitoring nutritional status in children have been made in the last decade. The 2014 consensus statement of the Academy of Nutrition and Dietetics and American Society for Parenteral and Enteral Nutrition (ASPEN) recommends the use of anthropometric Z‐score cut‐offs and mid‐upper arm circumference (MUAC) to identify malnutrition in children if only a single data point is available, or weight gain velocity (<2 years of age), weight loss (2–20 years of age), deceleration in weight‐for‐length or height Z‐score and inadequate nutrient intake (% of estimated energy/protein needs) if multiple data points are available.^21^ They also suggest cut‐offs for diagnosing mild, moderate and severe malnutrition. These guidelines refer to children in general, and not to children with SNI,^21^ and notably, Z‐score cut‐offs are poor indicators of malnutrition in children with CP.^22^ The 2017 European Society for Paediatric Gastroenterology, Hepatology and Nutrition (ESPGHAN) guidelines recommend 6‐monthly assessment using multiple methodologies, including height, weight, skinfold thickness and MUAC in children with neurological impairment.^23^ A toolkit for healthcare professionals, which presents decision trees for alternative methods of obtaining height and weight measurement in children with CP when direct measurement is not possible, has been proposed.^24^ This includes recently developed equations for weight estimation in children with CP using MUAC, GMFCS level and age.^25^ However, although accurate at the population level and useful in research or public health efforts, at present, their utility on the individual level is unclear.^26^

Both the ASPEN and ESPGHAN guidelines recommend utilising reference data from the general population, such as the World Health Organization or the Centres for Disease Control and Prevention.^21^, ^23^ Differences in body composition of children with SNI may suggest the use of a diagnosis‐specific growth reference, as commonly used in specific genetic conditions such as Down syndrome,^27^ Cornelia/Brachman de Lange syndrome^28^ and Rett syndrome.^29^ However, whether these indicate optimal growth or simply describe growth is a controversy that needs to be studied further. Growth charts based on a population of children with CP stratified for functional severity (GMFCS level) and feeding method (gastrostomy) for both boys and girls were initially published in 2007^30^ and refined and re‐published in 2011.^31^ Charts for weight‐for‐age, height‐for‐age and body mass index (BMI)‐for‐age are available, but due to uncertainty around the reliability of height measurement in the CP population, the authors recommend interpreting the height and BMI charts with caution.^30^ The unique value of the weight‐for‐age chart comes from the identification of a threshold for low weight, which is associated with greater morbidity and mortality.^31^ Children with a weight‐for‐age below the 20th percentile were more likely to have medical comorbidities (*p* < 0.01), and children classified as GMFCS level III, IV, V or V, and fed via a gastrostomy tube, who were below the 20th percentile, had a significantly increased risk of premature mortality (hazard ratio = 1.5, 95% confidence interval [CI] 1.4–1.7).^31^ The strengths of these charts are that they are based on more than 100,000 measurements from over 25,000 children over a 14‐year period, and they are based solely on weight, which can be obtained reliably on most children with CP.^31^ Whereas the ASPEN guidelines did not mention CP‐specific growth charts, the ESPGHAN guidelines recommend against their use.^23^ Authors argued that clinicians needed a growth chart that indicates ‘ideal’ growth based on a standardised reference population, such as the World Health Organization growth charts, where co‐occurring conditions such as malnutrition have not affected growth patterns.^23^ On the contrary, the differences in body composition and difficulties with obtaining accurate height measurements among children with CP, particularly those at the severe end of the spectrum, make standard growth charts challenging and somewhat subjective to apply. The association of a reliable growth indicator (i.e., severity‐specific weight percentile) with important health outcomes, such as major medical conditions and mortality, suggests that the CP growth charts may deserve further study.

### The challenge in measuring body composition

Due to altered growth and body composition in children with SNI, accurately assessing fat and muscle stores is especially problematic using indirect measures such as BMI. The use of BMI categories in children with CP frequently misclassified children as ‘normal weight’ or ‘underweight’, whereas a gold standard measure of body composition, such as dual‐energy X‐ray absorptiometry (DXA), showed they had either excess or adequate fat stores relative to normative ranges.^32^ The need for nutritional intervention in a child with SNI should therefore not be determined based solely on BMI. Body fat percentage should also be used with caution in guiding nutritional intervention in children with SNI. A child with SNI is likely to have lower lean mass than children with typical development; therefore even if they have the same amount of fat mass, the child with SNI will have a higher body fat percentage than the child with typical development due to their overall lower body weight.^33^ Attempts to attain a ‘healthy’ body fat percentage will effectively reduce their overall nutritional reserves even further.

A recent systematic review of assessment methods to estimate body composition in children with CP highlighted that, although measures such as bioelectrical impedance (BIA), skinfolds and associated body fat estimation equations have demonstrated validity on a population level,^34^, ^35^ they have poor validity for assessing body composition in an individual child.^36^ To assess if a child with CP is in positive or negative energy balance over time, *raw data* from skinfold measurements (thickness in mm) or BIA measurement (impedance in Ohm) could be used instead and in conjunction with weight and other measures to guide nutritional management.^36^, ^37^

### Estimating energy requirements

Although many researchers have attempted to develop formulations for estimating resting energy expenditure in children with CP, all are inaccurate on an individual level.^38^ The energy requirements of children and adolescents with CP who utilise a wheelchair for mobility are significantly lower than those of healthy, typically developing children or ambulatory children with CP (GMFCS level I–II). However, there is no evidence to suggest protein or micronutrient needs are lower.^39^, ^40^, ^41^, ^42^ Providing adequate nutrients in the context of a lower energy requirements becomes a challenge, particularly for orally fed children with SNI and children who receive blended tube feeds. Although this challenge may be considerably easier to overcome with commercially formulated tube feeds, issues around tolerance of these feeds in terms of gastrointestinal symptoms may occur. Irrespective of mode of feeding, regular monitoring of nutritional status via growth, body composition and micronutrient status remains critical in establishing energy requirements and nutrient needs to ensure children are not over‐ or undernourished.

### Paediatric Subjective Global Nutrition Assessment

In children with SNI, monitoring nutrition status and identifying those at risk of becoming malnourished is often a challenge. Although poor growth may be an indicator of poor dietary intake, children with SNI also tend to grow differently due to their reduced mobility and other factors related to the underlying disorder. The Paediatric Subjective Global Nutrition Assessment (SGNA) is a comprehensive, organised method of assessing nutritional status and has been gaining traction for use in children with CP.^43^, ^44^, ^45^ To complete the SGNA, a dietitian performs a structured nutrition‐focused physical examination (loss of subcutaneous fat, muscle wasting, oedema), collects a nutrition‐focused medical history (linear growth, weight‐for‐height/length, changes in body weight, adequacy of dietary intake, persistent gastrointestinal symptoms, functional impairment, metabolic stress) and assigns a global classification of ‘well nourished’, ‘moderately malnourished’ and ‘severely malnourished’.^46^ The SGNA has been found to perform better than objective anthropometric and biochemical measures for predicting risk of infectious complications and longer hospital stays; however, more research is needed to link these measures with clinically significant outcomes.^47^ Importantly, malnutrition is increasingly understood as a continuum, and although cut‐off such as weight‐for‐age Z‐score under −2 or triceps skinfold thickness <10th centile for age and sex can alert cases already malnourished and in need of intervention, regular monitoring may highlight inadequacy in nutrition prior to reaching a status of ‘malnourished’.

### Eating and Drinking Ability Classification System

The risk of poor nutritional status is inextricably linked to a child's feeding difficulty. The Eating and Drinking Ability Classification System (EDACS) provides a common language between people with CP, their parents, healthcare professionals and inter‐agency,^48^, ^49^ and can be used in prognosis and service planning. The EDACS classifies children's regular mealtime performance according to safety (choking and aspiration risk) and efficiency (time taken in relation to peers, and loss of food and fluid from the mouth). These may be influenced by limitations to oropharyngeal sensorimotor functions required for sucking, biting, chewing, oral transit and swallowing.^50^ It classifies the child's eating and drinking ability on a 5‐point scale (I–V), and the level of assistance required at mealtimes on a separate 3‐point scale.^50^ The original tool is valid from 3 years of age to adulthood, while the Mini‐EDACS is for those aged 18–35 months.^50^, ^51^, ^52^ Both parent and therapists can report a child's EDACS level, and it is used an adjunct to comprehensive assessment of feeding skills, including instrumental (e.g., video fluoroscopic swallowing studies, fibreoptic endoscopic evaluation of swallowing, manometry) and clinical (e.g., dysphagia disorder survey).^50^, ^51^, ^52^

### Feeding and Nutrition Screening Tool

Screening tools can help medical and allied health professionals quickly identify children who may benefit from further nutrition and feeding assessments. A 2021 scoping review identified one recently developed validated screening tool which uses a combination of four questions with high sensitivity and specificity for identifying children with CP with undernutrition and feeding/swallowing difficulties when compared to the EDACS and/or video fluoroscopic swallow studies and the SGNA.^53^, ^54^ The Feeding and Nutrition Screening Tool (FNST) can be used in children from the age of 2 years, and the four questions are as follows: ‘Do you think your child is underweight?’; ‘Does your child have problems gaining weight?’; ‘Rate on a scale from 0 to 10, whether you think your child has any problems eating compared to other children of their age?’; and ‘Rate on a scale from 0 to 10, whether you think your child has any problems drinking compared to other children of their age?’.^53^ Answering ‘yes’ or ‘unsure’ to the first two questions or marking ≥7 on the visual‐analogue scale for the last two questions receives a score of one point each. If the total score is ≥3, the child is flagged for further assessment of feeding/swallowing and nutritional status.^53^ It is important to note that the tool is designed to be used in an outpatient setting in a population of individuals with CP with a high prevalence of chronic undernutrition and feeding/swallowing difficulties, and not in an inpatient setting with acutely unwell children.^53^

## NUTRITIONAL MANAGEMENT

### Gastrointestinal symptoms and the gut microbiome

There is an increased understanding of the importance of specific types of dietary fibre on the gut microbiome and their effects on gastrointestinal function.^55^, ^56^, ^57^ A significant proportion of children with SNI experience gastrointestinal symptoms such as gastro‐oesophageal reflux disease (GORD), gastrointestinal dysmotility, vomiting, diarrhoea, constipation and feed intolerance, some of which are associated with the use of anti‐epileptic medication,^58^ others a consequence of central nervous system damage, over‐feeding, low mobility, scoliosis and positioning.^8^, ^59^ Children with SNI on long‐term enteral feeds have been found to have different and less favourable gut microbiome taxonomy relative to age‐ and sex‐matched children with typical development.^60^, ^61^ The diversity of the gut microbiome appears to be influenced by the diversity of the human diet, especially the consumption of a variety of plant‐based foods; meanwhile a diet solely of commercial enteral feeds would provide limited, if any, diversity.^62^

Guidelines for the management of constipation in children recommend ‘adequate fibre’ in combination with laxatives, adequate fluid intake and age‐appropriate physical activity, but despite the known benefits of diverse types of fibre, no recommendation is made with regard to the source or type of dietary fibre. Dietary fibre may be described in terms of its physicochemical properties (solubility, viscosity) as well as its physiological effects (fermentability, bulking).^63^, ^64^ Different combinations of properties have differing functional or physiological effects within the human gut, and they influence micronutrient availability, gut transit time, stool formation (bulking and water‐holding) and microbial specificity.^63^ In terms of the use of fibre in the management of gastrointestinal symptoms, soluble, viscous fibre with a high water‐holding capacity and resistance to fermentation (e.g., psyllium) can aid symptoms of diarrhoea by aiding stool formation and slowing down transit time, as well as symptoms of constipation by softening stools.^63^ Although fermentable fibres may theoretically add to stool weight by increasing bacterial biomass, the bulking effect of (predominantly) insoluble, non‐viscous fibres (e.g., coarse wheat bran) has a more significant impact on stool consistency and stool weight and improved transit time via the mechanical stimulation of the intestinal mucosa.^63^ Further research is needed to determine the ideal dosing and mix of fibres to recommend, and the impact of food processing (e.g., blending) on the properties of different fibres, as the bulking effect of insoluble fibres relies on the structure remaining intact.^65^

Fibre was omitted from early commercial enteral formulations due to concerns about increased viscosity and sedimentation.^66^ Although these issues have largely been overcome, many of the commonly used commercial feeds today only contain small amounts of fibre. There is a limited amount of research available assessing the use of fibre‐containing enteral feeds in children with SNI, but findings are generally positive.^64^, ^67^, ^68^, ^69^, ^70^ The optimal amount, type and combinations of fibre in enteral feeds are still to be determined for these children and will depend on the child's symptoms. Manufacturers of commercial enteral feeds are well placed to collaborate with researchers to further our knowledge in this area and offer a wider variety and amount of fibre‐containing enteral feeds.

### Tube feeding

The most significant change in the nutrition management of children with SNI over the past decade has arguably been the increased focus on the use of blended food tube feeds. The British Dietetic Association published a policy statement on the use of blended food in enteral feeding in 2019.^71^ Although the previous 2013 policy suggested dietitians should support patients who chose to use blended food tube feeds, the updated guidelines suggest that blended food tube feeds be offered *as a choice* to patients and carers by the dietitian. This change came about due to advocating a shared decision‐making approach in accordance with the National Institute for Healh and Care Excellence,^72^ as opposed to a risk assessment approach.^73^ In 2021, the British Dietetic Association published a Practice Toolkit to support dietitians in managing patients on blended food tube feeds.^74^ The Australasian Society for Parenteral and Enteral Nutrition (AusPEN) published a consensus statement on blended food tube feedings in 2021,^75^ and the ESPGHAN Committees of Allied Health Professionals and Nutrition published a comprehensive guide for managing blended food tube feeds in 2023.^76^ Dietitians, in collaboration with the child's treating team and caregivers, at early‐adopter hospitals now initiate blended food tube feeds as soon as the stoma has healed (approximately 3 months post‐surgery) in patients where it is found to be an appropriate option.^77^

The return of blended food tube feeds represents a full‐circle moment, as the use of pureed foods was first established for those with a feeding disability in ancient Greece and Egypt.^78^ The resurgence of blended food tube feeds has primarily been driven by parental demand and perhaps the general cultural shift of favouring more ‘whole’ and ‘unprocessed’ food.^79^ Prolonged and stressful mealtimes are frequently reported by parents of children with SNI, and parents also report spending a significant amount of time preparing safe foods and drinks for their children and managing tube feeds.^80^, ^81^, ^82^ For families, providing sustenance to their child is not just the provision of energy and nutrients; it also has social‐emotional significance. Enteral feeding has been reported to create a sense of distance.^82^, ^83^ Therefore, providing the family meal in a blend may help bridge this sense of disconnect. Families and researchers have also reported benefits of blended food tube feeds in the management of gastrointestinal symptoms such as vomiting, retching, diarrhoea and constipation, but a greater volume of data are needed to confirm these observations.^84^, ^85^, ^86^ The first prospective study of transitioning medically complex children who received >75% of their energy requirements from commercial enteral feeds to a blended food diet demonstrated that it was both safe and well tolerated, but to maintain BMI Z‐scores, a higher energy diet (1.5‐fold increase in kilojoules) was required, which needs to be explored further.^87^ Importantly, caregivers reported very high satisfaction with the use of a blended food diet, despite the increased cost and time commitment.^87^

Due to the labour‐intensiveness of blended food tube feeds, a reasonable approach may be to provide it at some meals only; and a recent, but small study found that children who received at least 25% of their energy from blended food had significantly fewer gastrointestinal symptoms than those receiving commercial tube feeds.^88^ This study also reported that children who were exclusively fed with blended foods had a significantly lower weight Z‐score (−1.7 ± 2.1 vs. −0.5 ± 1.4) and BMI Z‐score (−0.6 ± 2.1 vs. 0.4 ± 0.8) than those who were fed commercial tube feeds. However, these differences were not observed when these two methods were combined, that is, using both blended food and commercial feeds.^88^ Larger, prospective studies using reliable measures of body composition are needed to confirm these findings.

With greater acceptance and promotion of blended food tube feeds in healthcare settings, there may be room for the development of complimentary products to simplify the process and make personalised prescriptions depending on a child's needs, including specific vitamin and mineral supplements and shelf‐stable blended food tube feeds for outside the home, for example, when travelling or attending social events. Importantly, there is a need for easily accessible, credible information on making blends that meet nutritional needs, as well as guidance on the more complex hygiene and food safety aspect of providing blended food tube feeds.

### Gastrointestinal dystonia

Children with SNI can also experience an array of serious gastrointestinal symptoms beyond those of reflux, constipation or dependence on enteral tube feeds. For some, enteral tube feeding leads to disabling dystonia (i.e., involuntary muscle contractions that cause repetitive or twisting movements), coined ‘gastrointestinal dystonia of severe neurodisability’, or GID. A working definition of GID was proposed in 2022: *‘Clinical manifestations of distress (pain behaviour, hypertonicity, retching, vomiting, vagal phenomenon, abdominal distention) attributable to the gastro‐intestinal tract, directly and indirectly related to feeding and bowel habit, where confounding systems distress have been addressed or excluded’*.^89^ Key features include a GMFCS level IV or V (i.e., non‐ambulant) or equivalent, and a clear temporal relationship with feeding at some point in disease course. The severity of symptoms has resulted in significant malnutrition due to feed cessation and is the most significant factor in reducing current quality of life.^89^ Assessment and a positive clinical diagnosis by a paediatrician with specialist training in movement disorders, a paediatric gastroenterologist and a specialist paediatric nutrition support team are recommended for children experiencing GID.^89^

The most appropriate treatment options for children experiencing GID are still not fully understood.^90^, ^91^, ^92^ There is emerging evidence that tube feeds using blended food may help in GID, but further research is required.^93^ The Association for Paediatric Palliative Care has recently published guidelines for ‘gastrointestinal dystonia in children and young people within the palliative care setting’.^94^ This document highlights the intricacies of managing optimisation of nutrition, feeding, constipation, gastro‐oesophageal reflux and polypharmacy in this complex condition, and emphasises working with the child and the family to ensure their goals are a cornerstone in assessment and management. These guidelines recommend trialling blended food via tube prior to a trial of post‐pyloric feeding but emphasise that these decisions should be made in partnership with the child's caregivers.^94^

### Long‐term health: overnutrition and cardiometabolic disease

There is some evidence that children with CP across the spectrum of severity are at greater cardiometabolic risk than typically developing peers.^95^ Non‐ambulatory children with CP in high‐income countries have, on average, relatively less fat‐free mass and greater fat mass than children with typical development, though this may vary by whether they are fed orally or by tube.^35^, ^96^ Whereas some studies report that the prevalence of overweight and obesity is lower for children with CP relative to children with typical development,^97^ others report that it is higher,^98^ some report that it is increasing^99^ and others found no change over time.^98^ Most prevalence studies have, however, used BMI categories to classify weight status. As discussed, BMI is not a valid measure of body composition in children with CP.

What is known is that adults with CP are at greater risk of morbidity and mortality from non‐communicable diseases such as stroke, chronic obstructive pulmonary disease and other heart conditions relative to the general population.^100^ The causes for this are likely many, including altered body composition with greater levels of visceral fat, lower levels of cardiorespiratory endurance, muscle strength and habitual levels of physical activity, higher levels of sedentary behaviour, poorer diet quality and poorer sleep than typically developing peers.^101^ There is a need for population‐based, longitudinal studies using validated measures to identify risk factors for cardiometabolic disease for individuals with CP. A core set of outcome measure instruments to measure risk factors for cardiometabolic disease in CP has been developed, and the importance of the included health outcomes, and the feasibility of measurement, was assessed in participants with CP and their families.^102^, ^103^ This latter step highlights the increased focus on undertaking research which has saliency in the population to be studied.

## ORAL FEEDING

The mainstays of interventions for improving oral feeding and ingestion functions in children with SNI continue to emphasise positioning, oral appliances, oral stimulation, sensorimotor facilitation and caregiver training.^104^ The Focus on Early Eating and Drinking Swallowing (FEEDS) toolkit was developed in 2022 as a consensus‐based parent‐professional set of feeding intervention recommendations for children with neurodisability.^9^ A total of 19 interventions broadly categorised as (i) targeting physical aspects of the child's dysphagia, (ii) behavioural changes to parent or child and (iii) a focus on family well‐being were essential to the toolkit and should inform future clinical management and research priorities.

A major shift in focus for feeding management for children with SNI is targeting direct active interventions during the infancy period (<12 months). In the recent Clinical Practice Guideline for early intervention for infants at risk of CP based on nine systematic reviews, only two interventions were recommended for developing eating and drinking skills for children with dysphagia: soft food (conditional recommendation) and a semi‐reclined/upright position (conditional recommendation).^105^ Other interventions lack sufficient high‐quality evidence to provide conclusive positive or negative recommendations. Published after this clinical practice guideline, babiEAT is a new individualised speech pathologist‐delivered intervention based on the principles of motor learning and neuroplasticity, which consists of 16 sessions delivered over 3 months.^106^ It was tested in a pilot randomised controlled trial (RCT) of 14 infants at risk of CP with dysphagia (mean age 9.5 months) and concluded that babiEAT infants showed significantly greater efficiency in fluid intake, fewer compensatory strategies with cup drinking, more complex food textures, and shorter mealtimes, and no associated adverse outcomes; however, the participants’ neurological severity was not described.^106^ Further, the newly developed Functional Chewing Training (FuCT) programme was recently subject of a systematic review, identifying three efficacy RCTs (total *n* = 168).^107^ Parents receive training by a physiotherapist and deliver the FuCT for 12 weeks to improve chewing function and reduce severity of tongue thrust and drooling in children with CP (birth to 18 years, with half of their research participants being GMFCS level IV–V). The intervention modifies the position of the child, adjusts food placement, provides sensory stimulation, incorporates chewing exercises and adjusts food consistency. The systematic review concluded that FuCT interventions consistently demonstrated improved chewing function but inconsistently (in two of three trials) demonstrated improvements to tongue thrust and drooling severity.^107^

Research continues in the fields of oral‐sensorimotor interventions,^108^, ^109^ neuromuscular stimulation^110^, ^111^ and co‐application of neurodevelopmental therapy with feeding interventions.^112^ A continued challenge to incorporating these interventions into clinical practice are limited sample sizes and limitations to the methodological rigour of the research.

### Risk feeding/eating and drinking with acknowledged risk

In some exceptional cases, including children with irreversible but nonprogressive severe disability and those with severe GID (‘life‐limiting conditions’), ‘risk feeding’ or ‘eating and drinking with acknowledged risk’ may be considered. ‘Risk feeding’, more commonly applied in the adult palliative care context, describes the choice to continue eating or drinking orally despite the known risks of aspiration or choking.^113^ Reasons for caregivers choosing risk feeding may include a child not tolerating tube feeding,^114^ quality‐of‐life aspects, religious/spiritual, cultural or related to the parent's perceived significance of feeding for parent‐child attachment.^115^ Decisions should be made considering the course of the illness (medically stable or deteriorating/terminal) and ‘real’ versus ‘assumed’ risks resulting in harm based on clinical indicators, such as choking risks, reduced oxygen saturation while feeding, child distress during mealtimes and the life‐threatening respiratory sequelae.^116^ A plan should be developed in consultation and collaboration with the family, treating physician and interprofessional team, with a focus on harm minimisation strategies to reduce ‘assumed risks’ including specific triggers and thresholds for review.^116^

## RECENT CHANGES IN MEDICAL MANAGEMENT THAT IMPACT ON NUTRITIONAL CARE

### Spasticity and dystonia

Over 80% of children with CP experience spasticity, a form of muscle overactivity characterised by a velocity‐dependent increase in muscle tone.^117^ It was previously thought that spasticity increases energy expenditure, though evidence suggests that this is not the case, rather fat‐free mass and ambulatory status have significant associations with energy requirements.^42^ Further, children who underwent selective dorsal rhizotomy and had significant reduction in spasticity did not show reduced energy consumption relative to a control group of children with CP who did not undergo treatment for spasticity.^118^

Dystonia is a movement disorder in which involuntary sustained or intermittent muscle contractions cause twisting and repetitive movements, abnormal postures or both.^119^ Dystonia is the primary movement pattern in dyskinetic CP but can also be present alongside spasticity. Dystonia is thought to cause a relative increase in energy expenditure, and a significant reduction in symptoms through medical management may reduce energy requirements, but evidence is limited and needs to be reviewed on a case‐by‐case basis.^120^

### Gastrointestinal symptoms

The use of proton pump inhibitors (PPI) to manage GORD in children has seen a significant increase across many health systems in the past decade^121^, ^122^, ^123^, ^124^ and are often regarded as safe, even for prolonged periods of time.^125^ Some evidence suggests that that PPIs alter the gastrointestinal microbiome in children, possibly through lowering the acidity of the gut.^126^ Gastrointestinal infection caused by clostridium difficile has also been linked to PPI use.^127^ Further, there is increasing awareness that PPIs can be associated with an increased risk of bone fractures in children.^128^ This risk is small but significant^128^ and may be linked to reduced calcium absorption.^129^

## CONCLUSION

The last decade has seen the emergence of assessments and classification tools and guidelines for the nutritional management of children with SNI. A better understanding of the importance of fibre and the gut microbiome has emerged, and although consequences are not fully known, commercial tube feeds appear to alter children's gut microbiome. A new diagnosis related to severe feeding intolerance has been defined, but the medical treatment of gastrointestinal symptoms has seen relatively little progress in the last decade. Due to earlier diagnosis, early oral feeding interventions based on motor‐learning principles have emerged. The greatest change in the field, however, is arguably the resurgence of blended food tube feeds, primarily driven by parent demand. It holds promise as a tool to reduce complications and concerns associated with commercial feeds, both in terms of gastrointestinal symptoms and the parent‐child relationship.

Due to their altered growth patterns relative to reference norms, a persistent challenge remains in identifying undernourished and deteriorating children versus those who are growing differently due to their neurological impairment and reduced mobility but are otherwise healthy. A cornerstone of nutritional management therefore continues to include careful tracking of multiple anthropometric measures, but consensus on how to best monitor and track nutritional status is still needed. As the life expectancy of people with SNI has increased due to advances in medical and nutrition care, our approach needs to include a view to long‐term health outcomes. A balance between ensuring adequate nutrition to support growth, development and well‐being, but avoiding overnutrition and associated detrimental long‐term effects on cardiometabolic health is therefore key in the nutritional care of this population.

## AUTHOR CONTRIBUTIONS

All authors made an active contribution to the design, analysis, interpretation of the findings and drafting of the paper. All authors critically reviewed its content and approved the final version submitted for publication.

## CONFLICT OF INTEREST STATEMENT

The authors declare no conflicts of interest.

### PEER REVIEW

The peer review history for this article is available at https://www.webofscience.com/api/gateway/wos/peer-review/10.1111/jhn.13277

## Data Availability

Data sharing is not applicable to this article, as no new data were created or analysed in this study.
